# *Talaromyces marinus* (Eurotiales, Trichocomaceae): a new marine fungus from the Red Sea, Egypt, with distinguished enzymatic capabilities

**DOI:** 10.1186/s12866-026-05566-8

**Published:** 2026-09-07

**Authors:** Ahmed Elbadry Abdel-Aziz, Enas Mahmoud Amer, Mahmoud Saadeldin Bakhit

**Affiliations:** 1 Botany and Microbiology Department, Faculty of Science, New Valley University, El-Kharga, 72511 Egypt; 2 Botany and Microbiology Department, Faculty of Science, Assiut University, Assiut, 71515 Egypt; 3 Botany and Microbiology Department, Faculty of Science, Sohag University, Sohag, 82524 Egypt

**Keywords:** Eurotiomycetes, Intertidal fungi, Saprobes, Mangrove, Molecular phylogeny

## Abstract

**Background:**

*Talaromyces* C. R. Benj., is a large and taxonomically important genus within Ascomycota, established to accommodate the teleomorphs of certain *Penicillium* Link, species and is now recognized as a monophyletic lineage distinct from *Penicillium*. Multigene phylogenetic analyses demonstrated that species formerly placed in *Penicillium* subgenus *Biverticillium* belong within *Talaromyces*, leading to their reclassification under the single-name nomenclature system. Currently, the genus comprises 237 species arranged into nine sections supported by multi-locus phylogeny. Species of *Talaromyces* are cosmopolitan, occurring in diverse habitats and substrates. It is notable for their medical, industrial, and ecological significance. In Egypt, several species of *Talaromyces* have been reported from a variety of substrates, highlighting the regional diversity and ecological relevance of the genus. Through the ongoing work a new *Talaromyces* species was isolated and identified.

**Methodology:**

Morphological characterization combined with multi-locus phylogenetic analyses based on ITS (internal transcribed spacers of rDNA), LSU (nuclear large subunit rDNA), *BenA* (β-tubulin), and *RPB2* (RNA polymerase II second largest subunit) loci were employed to accurately determine the systematic placement of the new species. In addition, the enzymatic potential of the proposed new taxon was evaluated using standard screening assays.

**Results:**

A new marine taxon, *Talaromyces marinus* is reported from decaying woody samples of *Avicennia marina* (Forssk.) Vierh., collected from Wadi El-Jimal area, Red Sea Governorate, Egypt. Multi-locus analyses of the combined ITS, LSU, *BenA*, and *RPB2* sequences dataset placed the new species within *Talaromyces* section *Trachyspermi* series *Diversi* but distinct from previously described species. The new taxon is characterized by restricted growth on different media, long stipes, biverticillate penicilli, the shortest acerose phialides in series *Diversi*, globose to subglobose or ellipsoidal, smooth-walled conidia, acid production, and soluble pigments.

**Conclusion:**

*Talaromyces marinus* sp. nov. is described, illustrated, phylogenetically analyzed, and its enzymatic potential is evaluated, it also compared with other species in the section *Trachyspermi* series *Diversi*.

**Supplementary Information:**

The online version contains supplementary material available at https://doi.org/10.1186/s12866-026-05566-8.

## Introduction

*Talaromyces* is one of the largest genera within the phylum Ascomycota. It was introduced by C.R. Benjamin to accommodate the teleomorph of certain *Penicillium* species, with *T. vermiculatus* (= *T. flavus*) as the type species. Its teleomorph is characterized by cleistothecial, soft, superficial, yellow ascocarp with a wall of interwoven hyphae, and globose to subglobose asci containing ornamented ascospores [1], whereas anamorph have biverticillate or rarely terverticillate conidiophores with acerose, narrow-mouthed phialides, and smooth-walled, globose to ellipsoidal conidia that formed in basipetal chains [2, 3].

According to the infrageneric classification system *Penicillium* was divided based on structure and branching patterns of the conidiophores into four subgenera: *Aspergilloides*, *Biverticillium*, *Furcatum*, and *Penicillium* [4]. Initial molecular analysis of *Penicillium* subg. *Biverticillium* demonstrated that these species were phylogenetically distinct from those classified under the other three subgenera [5, 6]. For many years, *Penicillium* was considered polyphyletic with species classified under the subgenus *Biverticillium*. However, multigene analyses suggested excluding these species from *Penicillium* [5]. Subsequent studies demonstrated that most species of the subgenus *Biverticillium* and *Talaromyces* cluster within a monophyletic clade [7, 8]. Several species of *Penicillium* subg. *Biverticillium* were reassigned to *Talaromyces* based on phylogenetic analyses of DNA sequences of the nuclear rDNA internal transcribed spacers (ITS) and the first large subunit of RNA polymerase (*RPB*1) [3]. Due to the single-name nomenclature concept, species from the subgenus *Biverticillium* were reclassified to be included in the genus *Talaromyces*, which incorporates both teleomorphic and anamorphic species [3].

Advances in molecular diagnostic techniques have led to the identification of 237 species within the genus *Talaromyces*, which are classified into nine sections: *Bacillispori*, *Helici*, *Islandici*, *Purpurei*,* Subinflati*, *Talaromyces*, *Trachyspermi*, *Tenues* and *Brunneospori* [2, 7, 9, 10]. Four gene phylogeny was used to support this sectional classification [9, 11, 12].

Among the nine sections, *Talaromyces* sec. *Talaromyces* comprises approximately half of the documented species within the genus [13, 14]. The second section in the species number is *Trachyspermi* that was established from *T. trachyspermus* based on differences in ubiquinone systems and the prevailing morphological classification [15]. Section *Trachyspermi* was restructured based on morphology and multi-locus datasets (ITS, *BenA*, *CaM* and *RPB2*) to include five new series, namely: *Diversi*, *Erythromelles*, *Miniolutei*,* Resinarum* and *Trachyspermi* [16]. Currently, 52 species are classified under section *Trachyspermi* [9, 16]. Species of this section are characterized by biverticillate conidiophores and cream white or yellowish ascomata when produced [17]. It also grows poorly on creatine sucrose agar (CREA), slightly faster on malt extract agar (MEA), restrictedly on Czapek’s yeast agar (CYA), yeast extract sucrose agar (YES), and dichloran 18% glycerol agar (DG18) [17].

Species of *Talaromyces* are cosmopolitan, distributed across temperate, tropical, and subtropical regions, and inhabit diverse substrates such as leaves, soil, fruits, wood, and house dust [2]. *Talaromyces* species hold significant medicinal, industrial, and ecological importance. However, several species are also known to cause infections in human and animals e.g. Talaromycosis [18]. While many are good producers of anticancer, antimicrobial compounds, enzymes, natural colorants, biocontrol agents against plant pathogens, antiproliferative and antioxidative compounds [19–28]. Recent studies demonstrate that *Talaromyces* species possess a strong capacity for biomass degradation, supported by genomic evidence of enzyme-encoding genes and experimental optimization of cellulase and xylanase production on lignocellulosic substrates [29–31].

Several *Talaromyces* species have been recorded from different substrates in Egypt [32]. Exploration of novel fungal species from marine habitat has gained increasing scientific interest due to their potential to produce bioactive secondary metabolites. Red Sea remains one of the least explored regions for the discovery and documentation of such fungi, so this study is considered one of the studies that introduce a new fungal species in *Talaromyces* to science.

## Materials & methods

### Samples collection

Intertidal decaying woody samples of *Avicennia marina* were collected during spring 2015 from a mangrove site in the Wadi El-Jimal area (24°40’35"N 35°05’14"E − 24°40’05"N 35°05’34"E) that is located 115 km south Marsa Alam at Red Sea Governorate, Egypt. Samples were brought to the laboratory in clean plastic bags. Metadata, including the date, geographic coordinates, and habitat were recorded on site. The samples were incubated in humid plastic boxes at 25 °C and examined periodically. Initial microscopic observations and examinations were made using an Olympus SZ61 (Olympus, Tokyo, Japan) stereomicroscope.

The collected plant materials of *A. marina* were identified by Dr. Ahmed Elkordy, Professor of plant taxonomy, Faculty of Science, Sohag University. Voucher specimens (SHG-2015-020) were deposited at Sohag University Botanical Herbarium (Institutional acronym: SHG) and is publicly accessible for verification.

### Isolation and morphological description

Pure cultures of the fungi were obtained by the single spore isolation method as described by Abdel-Wahab et al. [33]. Morphological characteristics were observed and described using standardized methods [34]. Eight standard growth media were used: Czapek’s yeast extract agar (CYA), Czapek’s agar (CZA), Malt extract agar (MEA), Oatmeal agar (OA) [3], Yeast extract sucrose agar (YES), Creatine agar (CREA) [35], Corn meal agar (CMA; HiMedia, BioSciences, India), and Potato dextrose agar (PDA; Oxoid, Basingstoke, England). The inoculum used was obtained from a 7 day-old pure isolate cultured on PDA. It was applied directly to the surface of each medium in 90 mm Petri dishes and then incubated in the dark at 25 °C for three weeks. Morphological characteristics of the colony, including growth rate, texture, obverse and reverse colors, production of soluble pigments, exudate production, and sporulation on all media, were determined and recorded after 7 days of incubation. Color names used in the descriptions were based on Rayner [36]. To determine the presence of the sexual morph, cultures on CYA and OA media were incubated at 25 °C in the dark for 40 days. Microscopic observations were performed on preparations made from cultures incubated on PDA for 2–3 weeks at 25 °C in the dark. Micromorphological features e.g. conidiophore, phialides, metulae and conidia were measured by using micrometer. Micrographs were captured using an Olympus BX51 compound microscope equipped with a Toup Tek XCAM1080PHA (Toup Tek, Zhejiang, China) digital imaging system. The shape, surface structure, size, conidia, metulae, and branching pattern of conidiophores were recorded. Dried specimens, slides, and cultures were deposited in Sohag University Microbial Culture Collection (SUMCC) under the numbers SUMCC H-15,029 (Holotype) and SUMCC 15,014 (ex-type).

### DNA extraction, PCR amplification and sequencing

Cultures grown on glucose, peptone and yeast (GPY) broth [37] were used for genomic DNA extraction using the Microbial DNA Extraction Kit (MOBIO; Mo Bio Laboratories, Carlsbad, CA, USA). The primer pairs ITS1/ITS4 [38], LR0R/LR7 [39], Bt2a/Bt2b [40] and 5 F/7CR [41] were used to amplify the DNA sequences of the internal transcribed spacers of rDNA (ITS), partial nuclear large subunit rDNA (LSU), β-tubulin (*BenA*), and RNA polymerase II second-largest subunit (*RPB2*), respectively. PCR amplification and sequencing were done as previously described [42] by Solgent Co. Ltd. (South Korea). The newly generated sequences were assembled using Sequencher 4.2.2 (Gene Codes Corporation).

### Molecular phylogenetic analysis

Assembled sequences were subjected to a BLASTn search to reveal the closest matches. The other sequences used in the analyses (Table 1) were selected following recent publications [16, 43, 44]. Sequences were aligned using ClustalX [45] and optimized manually. Single-locus and multi-loci aligned datasets were subjected to maximum likelihood (ML), maximum parsimony (MP), and Bayesian inference (BI) analyses. The MP analysis was performed using PAUP*4 [46]. ML analysis was conducted by RAxMLGUI v. 2.0.13 [47] with 1000 rapid bootstrap replicates under the GTR+GAMMA substitution model. BI analysis was performed with MrBayes v. 3.1.2 [48]. Phylogenetic analyses were performed based on details outlined previously [37, 49–51]. The resulting trees were viewed with NJplot [52], and the layout was created in Adobe Illustrator CC (Adobe Systems Inc., CA, USA). Newly generated sequences were deposited in NCBI GenBank.

**Table 1:** Taxa used in the phylogenetic analyses of *Talaromyces marinus*. The sequence generated in the study is indicated in bold

Species	Voucher/Strain number	Substrate	Country	GenBank accession no.			
				ITS	LSU	BenA	RPB2
*Talaromyces aerius*	CBS 140,611^T^	Indoor air	China	KU866647	—	KU866835	KU866991
*T. affinitatimellis*	CBS 143,840^T^	Honey	Spain	LT906543	—	LT906552	LT906546
*T. africanus*	CBS 147,340^T^	House dust	South Africa	OK339610	—	OK338782	OK338833
*T. albidus*	CGMCC 3.26143^T^	Soil	China	OQ746343	—	OQ746324	OQ746328
*T. albisclerotius*	CBS 141,839^T^	Soil	China	MN864276	—	MN863345	MN863334
*T. albobiverticillius*	CBS 133,440 ^T^	Decaying leaves	Taiwan	HQ605705	—	KF114778	KM023310
*T. amyrossmaniae*	NFCCI 1919^T^	Decaying fruits	India	MH909062	—	MH909064	MH909066
*T. assiutensis*	CBS 147.78^T^	Soil	Egypt	JN899323	MH872881	KJ865720	KM023305
*T. atroroseus*	CBS 133,442^T^	House dust	South Africa	KF114747	—	KF114789	KM023288
*T. austrocalifornicus*	CBS 644.95^T^	Soil	USA	JN899357	—	KJ865732	MN969147
*T. basipetosporus*	CBS 143,836 ^T^	Honey	Japan	LC763423	—	LT906563	LC763450
*T. brasiliensis*	URM 7618^T^	Honey	Brazil	MF278323	—	LT855560	MN969198
*T. calidominioluteus*	CBS 147,313^T^	Melon	Netherlands	OK339612	—	OK338786	OK338837
*T. catalonicus*	CBS 143,039^T^	Herbivore dung	Spain	LT899793	—	LT898318	LT899811
*T. chongqingensis*	CGMCC 3.20482^T^	Soil	China	MZ358001	—	MZ361343	MZ361357
*T. clemensii*	PPRI 26,753^T^	Wood in mine	South Africa	MK951940	MN388753	MK951833	MN418451
*T. convolutus*	CBS 100,537^T^	Soil	Nepal	JN899330	MT365179	KF114773	JN121414
*T. cystophilus*	CGMCC40002^T^	*Heterodera zeae* cyst	China: Guangxi	OM835900	—	ON164851	ON164852
*T. diversus*	CBS 320.48 ^T^	Mouldy leather	USA	KJ865740	MH867913	KJ865723	KM023285
*T. elephas*	CGMCC 3.28742^T^	Rotten husk of an unknown fruit	China: Yunnan	PV085756	—	PV102706	PV102727
*T. ellipsoideus*	CGMCC 3.22439	Sediment	China	OQ798985	—	OQ808981	OQ809036
*T. erythromellis*	CBS 644.80^T^	Soil	Australia	JN899383	MH877372	HQ156945	KM023290
*T. gaditanus*	CBS 169.81^T^	Air	Spain	MH861318	—	OK338775	OK338827
*T. germanicus*	CBS 147,314^T^	Indoor environment	Germany	OK339619	—	OK338799	OK338845
*T. guatemalensis*	CCF 6215^T^	Soil	Guatemala	MN322789	—	MN329687	MN329689
*T. hallidayae*	MST FP2569	Soil under turf grass	Australia	PP665728	—	PP682580	PP682567
*T*. *halophytorum*	KACC 48,127^T^	Roots of *Limonium tetragonum*	Republic of Korea	MH725786	—	MH729367	MK111427
*T. heiheensis*	HMAS 248,789^T^	Rotten wood	China	KX447526	—	KX447525	KX447529
*T. longistipes*	CGMCC 3.25509	Soil	China	OR680518	OR680585	OR843223	OR842935
*T. marinus*	**SUMCC 15,014** ^T^	**Decaying wood of** ***Avicennia marina***	**Egypt**	**PX683786**	**PX683787**	**PX694710**	**PX694709**
*T. mellisjaponici*	NBRC 116,048^T^	Honey	Japan	LC763421	—	LC763430	LC763448
*T. minioluteus*	CBS 642.68^T^	Unknown	Unknown	JN899346	MH867191	MN969409	JF417443
*T. minnesotensis*	CBS 142,381^T^	Human ear	USA	LT558966	—	LT559083	LT795605
*T. palmae*	CBS 442.88^T^	Seeds of *Chrysalidocarpus lutescens*	Netherlands	JN899396	MT365208	HQ156947	KM023300
*T. peaticola*	CGMCC 3.18620^T^	Soil (peat)	China	MF135613	—	MF284705	MF284704
*T. pernambucoensis*	URM 6894^T^	Soil,	Brazil	LR535947	—	LR535945	LR535948
*T. phialiformis*	CGMCC 3.22415^T^	Sediment	China	OQ798986	—	OQ808982	—
*T. phuphaphetensis*	TBRC 16,281^T^	Soil	Thailand	ON692803	—	ON706960	ON706964
*T. rubrifaciens*	CBS 140,498^T^	HVAC system	China	KR855658	—	KR855648	KR855663
*T. resinae*	CBS 324.83^T^	Resin of *Eucalyptus tereticornis*	China	MT079858	—	MN969442	MN969221
*T. rubidus*	CGMCC 3.26142^T^	Soil	China	OQ746342	—	OQ746323	OQ746327
*T. samsonii*	CBS 137.84^T^	Apple damaged by insect	Spain	MH861709	MH873419	OK338798	OK338844
*T. satunensis*	TBRC 16,246^T^	Soil	Thailand	ON692804	—	ON706961	—
*T. solicola*	DAOM 241,015^T^	Soil	South Africa	FJ160264	—	GU385731	KM023295
*T. speluncarum*	CBS 143,844^T^	Sparkling wine	Spain	LT985890	LS453298	LT985901	LT985911
*T. subericola*	CBS 144,322^T^	Sparkling wine	Spain	LT985888	NG075221	LT985899	LT985909
*T. subinflatus*	CBS 652.95^T^	Soil	Japan	JN899397	MT365211	MK450890	KM023308
*T. systylus*	BAFCcult 3419^T^	Soil	Argentina	KP026917	—	KR233838	—
*T. tianshanicus*	CGMCC 3.28741^T^	Soil	Uzbekistan	PV085759	—	PV102709	PV102730
*T. tianshanicus*	UZ08-27	Soil	Uzbekistan	PV085760	—	PV102710	—
*T. trachyspermus*	CBS 373.48^T^	Diseased fruits	USA	JN899354	MH867950	KF114803	JF417432
*T. ucrainicus*	CBS 162.67 ^T^	Potato starch	Ukraine	JN899394	NG064058	KF114771	KM023289
*T. udagawae*	CBS 579.72^T^	Soil	Japan	JN899350	—	KF114796	MN969148
*T. xishuangbannaensis*	CGMCC 3.28743^T^	Rotten husk of an unidentified fruit	China: Yunnan	PV085761	—	PV102711	PV102731

the new taxon highlighted by bold

^T^ refers to type strain

### Enzyme-producing potential

Enzymes production capabilities of the new species *T. marinus* were assessed using standard screening assays. Twenty qualitative plate assays were conducted to evaluate the production of the following enzymes: Asparaginase, Cellulase, Chitinase, Gelatinase, Inulinase, Laccase, Ligninase, Lignin peroxidase, Lipase, Manganese peroxidase, Tannase, Tyrosinase, and Xylanase. Agar plate assay technique was employed for screening of asparaginase production according to Theantana et al. [53]. The ability of *T*. *marinus* to produce cellulase was tested according to the method of Kasana et al. [54]. The method of Pasqualetti et al. [55] was followed for detecting Chitinase production. Screening for the production of Gelatinase and Inulinase followed the methods applied by Muslim et al. [56], while the production of Laccase and Tannase were tested according to D’Souza et al. [57] and Kumar et al. [58], respectively. Guaiacol supplemented agar was prepared according to D’Souza et al. [57] for detection of ligninase production. Media described by Atalla et al. [59] and Sivakami et al. [60] was used to screen the activity of Lignin peroxidase. For the detection of lipase production, minimal salt agar medium was prepared with olive oil as a sole carbon source, with slight modifications. The formation of a white precipitate was recorded as a positive result [61]. PDA medium containing 0.0025% phenol red (w/v) was employed [62] for manganese peroxidase production. Hydrolytic and oxidative activities of tyrosinase were screened using tyrosine screening medium, following the method described by Raval et al. [63]. Xylanase activity was detected according to the methods of Bailey et al. [64].

## Results

### Phylogenetic analysis

**Fig. 1:**
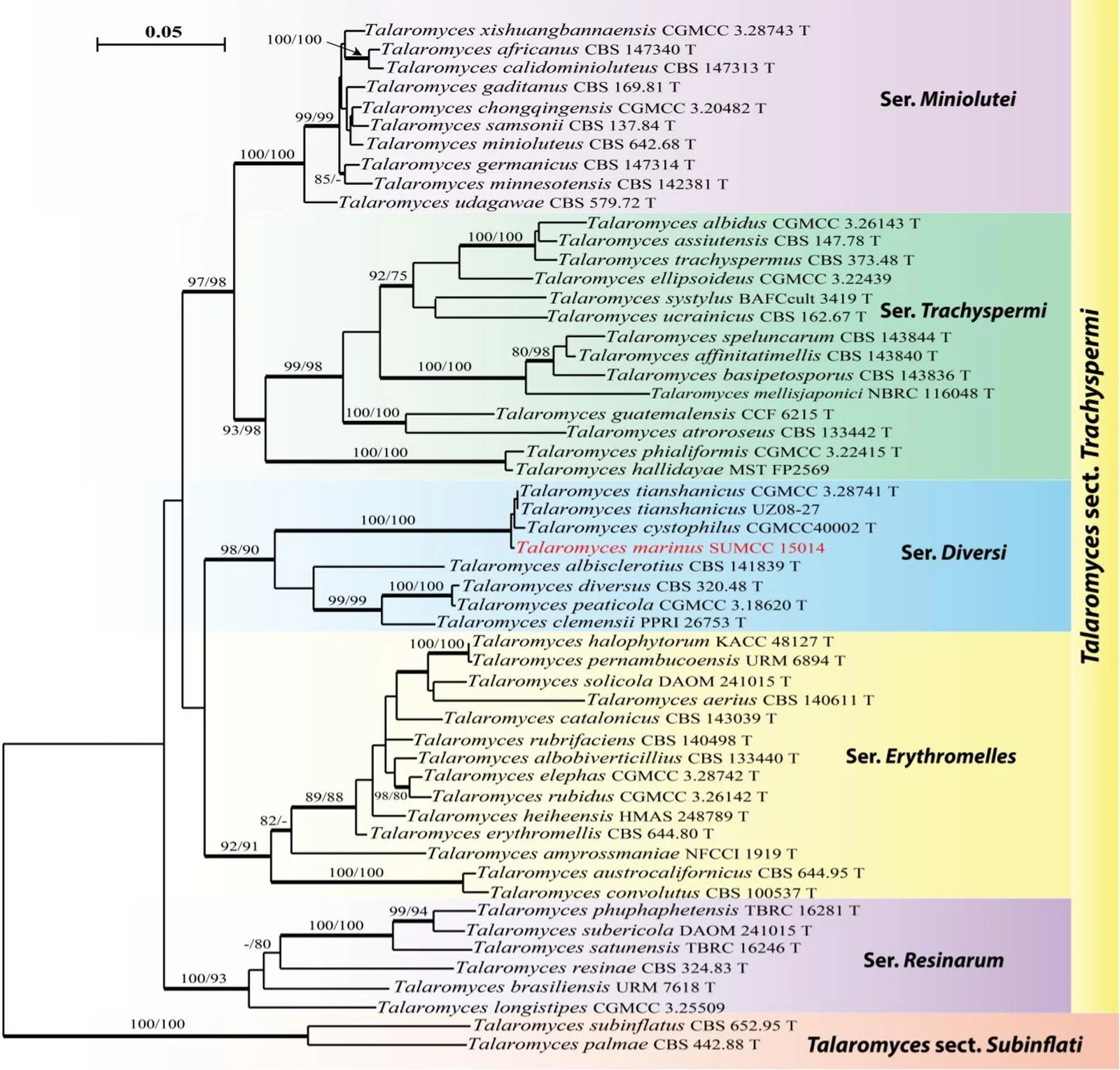
Phylogenetic tree generated from ML analysis (RAxML) based on a combined ITS, LSU *BenA*, and *RPB2* dataset sequences of *Talaromyces* sect. *Trachyspermi*. ML and MP bootstrap supports (≥ 70%) are indicated around the nodes. Branches received Bayesian pp ≥ 0.95 are in bold. The newly generated sequences are indicated in red

The concatenated ITS, LSU, *Ben*A, and *RPB2* sequence dataset encompassed 54 fungal strains (Table 1), of which 52 belong to *Talaromyces* section *Trachyspermi*, with *T. palmae* (CBS 442.88) and *T. subinflatus* (CBS 652.95) in *Talaromyces* section *Subinflati* as the outgroup. Detailed characteristics of the datasets are presented in Table (1). The most parsimonious tree was with a tree length of 2,646 steps (CI = 0.4512, RI = 0.7092, RC = 0.3200, HI = 0.5919). The ML analysis of the combined dataset yielded the best-scoring tree (Fig. 1) with a final ML optimization likelihood value of -22314.031986. The matrix had 1,276 distinct patterns with 33.90% completely undetermined characters and gaps. Estimated base frequencies were A = 0.253631, C = 0.259278, G = 0.235614, T = 0.251477; substitution rates, AC 1.477576, AG = 4.283453, AT = 1.041284, CG = 1.121222, CT = 6.154256, GT = 1.0; gamma distribution shape parameter α = 0.996930. The Bayesian analysis resulted in 30,000 trees after three million generations. The final average standard deviation of split frequencies was 0.004281. The tree topologies of ML, MP and BI did not show significant differences. Phylogenetic analyses of the combined ITS, LSU, *BenA*, and *RPB2* sequence data placed the new taxon, *T. marinus* (SUMCC 15014) in ser. *Diversi* within *Talaromyces* sect. *Trachyspermi* but distinct from previously described species (Fig. 1). The new fungus formed a basal clade to a node containing *Talaromyces cystophilus* Y.X. Mo & H.Y. Wu and *T*. *tianshanicus* X.C. Wang, L.Y. Peng, Gafforov & W.Y. Zhuang with significant statistical support (100/100/1.00 for ML/MP/BYPP, respectively). The single-locus datasets (ITS, *BenA*, and *RPB2*) were also compiled and analyzed to compare the topology and clade stability with those from the combined gene analyses, however these were not significantly different. The phylogenetic trees based on each single locus are presented in the supplemented data (Figures S1–S3).

### Taxonomy

*Talaromyces marinus* A.E. Abdel-Aziz and Bakhit sp. nov.

MycoBank: MB861560.

#### Typification:

Egypt, Red Sea (24°40’35"N 35°05’14"E − 24°40’05"N 35°05’34"E), isolated from intertidal decaying wood of *Avicennia marina* (Acanthaceae), 5 May 2015, coll. Ahmed E. Abdel-Aziz (Holotype, SUMCC H-15029), ex-type = SUMCC 15,014.

*Etymology*: *marinus* (Latin), referring to the sea where the species was collected.

In: *Talaromyces* sec. *Trachyspermi* sec. *Diversi*.

GenBank: ITS= PX683786, Partial LSU rDNA= PX683787, *BenA*= PX694710 and *RPB2* = PX694709.

### Macromorphology FIG. 2A-J

Colonies on CYA at 25 °C after 7 d: 19–25 mm diam., circular with regular margins, and convoluted at the center. The colony color was greenish yellow at the center and pale yellow toward the margin, while the reverse was brown in the middle and pale brown toward the margin. Exudate was present, but soluble pigment and sexual morph were absent. On YES at 25 °C after 7 d: 10–16 mm diam., irregular becoming velvety by the second week, surface velutinous; mycelium yellow to white at margins; reverse pale brown; exudate present; soluble pigment present. On MEA at 25 °C after 7 d: Colonies 8–16 mm diam., growing slowly, umbonate in central areas, depth of the colony moderate, margin entire; surface mealy; yellow and in some parts yellowish green and white toward the margins; reverse pale yellow; exudate present; soluble pigment present. On OA at 25 °C after 7 d: Colonies 5–8 mm diam, the growth is very weak, flat and looks like mucilage; entire margin; pale green color; reverse is yellowish green; no exudate; soluble pigment absent; sexual morph absent. On CZA at 25 °C after 7 d: Colonies 11–18 mm diam., irregular with entire margin, raised from the media surface, sulphur yellow color with white towards the margins, white reverse; exudate present, small yellow droplets; soluble pigment present. on CMA at 25 °C after 7 d: There is no detectable growth, while after 22 days 3–5 mm diam, yellow green color with irregular margins and reverse is yellow. On PDA at 25 °C after 7 d: 18–21 mm diam., floccose with entire margin, greenish at the center become yellow toward the margin, white to pale yellow reverse, exudate present; soluble pigment absent. On CREA at 25 °C after 7 d: there is no growth at the first week, after 14 days growing slowly and colonies about 6–9 mm diam.; deep yellow color; acid production present; soluble pigment absent.

### Micromorphology FIG. 2K-O

Conidiophores 112–200 × 2–3 μm (⁓ 150 × 2.5; *n* = 25), arising from aerial hyphae with long stipes, hyaline, septate, unbranched, smooth-walled to finely roughened, with slightly swollen apices, penicilli symmetrically biverticillate; Metulae 8–10 (–11) × 2.5–3 μm (*n* = 30); 4–6 per stipe, appressed, hyaline, cylindrical; Phialides 4–5 × 2–3 μm; 3–5 per metula, acerose, without long collula; Conidia globose to subglobose or sometimes ellipsoidal, 2–2.5 × 2.2–3 μm (⁓ 2.25 × 2.6 μm; *n* = 100), hyaline, smooth. Sexual morph not observed.

Notes: *Talaromyces marinus* is introduced as a new species based on morphology and phylogenetic analysis of combined ITS, LSU *BenA*, and *RPB2* sequence datasets. Molecular phylogenetic analyses placed the new taxon, *T. marinus* (SUMCC 15014) in ser. *Diversi* within *Talaromyces* sect. *Trachyspermi* in a clade containing the two species, *T. cystophilus* (CGMCC40002) and *T. tianshanicus* (CGMCC 3.28741 and UZ08-27). Morphologically, *T. marinus* exhibits all the diagnostic characteristics of the *Talaromyces* section *Trachyspermi* ser. *Diversi*. While it is distinguished from other species under series *Diversi* by the shortest phialides 4–5 × 2–3 μm and smooth to finely roughened conidiophore (Table 2). It also showed differences from the closest species *T*. *cystophilus* and *T*. *tianshanicus*. Notably, *T*. *marinus* has symmetrically biverticillate penicilli unlike *T*. *tianshanicus*, which possesses biverticillate, terverticillate or quaterverticillate penicilli [16, 65]. Additionally, the number of penicilli per metula ranges from 4 to 6, differing from *T*. *tianshanicus*, which is only 1 to 5 per metula. Metulae in *T*. *marinus* are appressed, similar to those in *T*. *cystophilus*, whereas they are divergent in other species within ser. *Diversi* [16, 65].

**Table 2:** Morphological comparison between *Talaromyces marinus* and their closely related species under sec. *Trachyspermi* ser. *Diversi*

Species	Colonies diam at 25 ℃, 7 d (mm)							Exudate	Soluble pigment	Conidiophore	Metulae	Phialide	Conidia	Sexual morph	Ref.
	CYA	YES	MEA	OA	CA	PDA	CREA								
*T. albisclerotius*	5–8	-	19–20	13–14	–	–	No growth	Absent, Except on OA present	Absent	70–130 × 3–4 μm, biverticillate, minor proportion having subterminal branches; stipes smooth.	8.5–11 × 4–4.5 μm, 3–5 per stipe, divergent	9–11 × 3–5 μm, 4–6 per metulae, acerose,	2–4.5 × 3–4 μm, subglobose to fusiform, smooth.	Absent, white sclerotia present on OA after 1 wk.	[10]
*T. clemensii*	5–8	6–7	30–31	0–11	–	–	6–7	Absent	Absent	150–520 × 3–4 μm, biverticillate, sometimes subterminal branched; Stipes smooth.	10.5–13 × 3–4 μm, 4–8 per stipe	10–12 × 2.5–3 μm, 4–6 per metula, acerose.	2–3 × 2–2.5 μm, broadly ellipsoid to ellipsoid, smooth.	Absent	[66]
*T. cystophilus*	21– 26	19–24	32– 33	18–22	–	–	–	On YES light-yellow droplets, on OA clear droplets	Absent	150–200 × 2.5–4 μm, biverticillate, stipes smooth.	8–13 × 2–3 μm, 4–8 per stipe, appressed.	10–12 × 1.5–2.5 μm, 1–4 per metula, acerose.	2.6–3.0 × 2.5–3 μm, subglobose to ellipsoidal, smooth.	Absent	[65]
*T. diversus*	–	–	After twelve to fourteen days 50–55	–	–	–	–	Absent	Absent	200–300 × 2–2.5 μm, symmetrical biverticillate, stipes smooth,	9–11 × 2–2.5 μm, 5–7 per stipe,	8–10 × 1.8–2.2 μm, 6–8 per stipe, compact clusters.	2–2.5 × 1.5–2 μm, elliptical to subglobose or broadly elliptical, smooth or delicately roughened.	Absent	[3]
*T*. *marinus*	**19–25**	**10–16**	**8–16**	**5–8**	**11–18**	**18–21**	**No growth in the first 7 days**, **After 14 days 6–9**	**Absent**,** Except small yellow droplets on CA**	**Clear zone around the colony on YES**,** CA and MEA.**	**112–200 × 2–3 μm**,** hyaline**,** septate**,** unbranched**,** smooth-walled to finely roughened**,** penicilli mostly symmetrically biverticillate.**	**8–10 × 2.5–3 μm**,** 4–6 per stipe**,** appressed**,** hyaline**,** cylindrical**	**4–5 × 2–3 μm; 3–5 per metulae**,** acerose**	**2–2.5 × 2.2–3 μm**,** subglobose to ellipsoidal**,** hyaline**,** smooth.**	**Absent**	**This study**
*T. peaticola*	5.0–6.9	9–11	24.7–24.9	11.7–12.5	–	–	3.3–3.9	Small clear droplets on MEA	Absent	160–200 × 3–4 μm, biverticillate, stipes smooth-walled.	7.5–11.5 × 2–3 μm, 3–8 per stipe, divergent.	7.0–13.5 × 1.5–2.0 μm, acerose,	1.5–2.5 × 1.5–2.0 μm, globose to subglobose, smooth.	Absent	[67]
*T. tianshanicus*	16–19	11–15	21–23	–	–	19–21	–	Absent	Absent	110–210 × 2.5–3.5 μm, irregularly biverticillate, terverticillate or quaterverticillate, stipes smooth-walled.	7–13 × 2.5–3.5 μm, 2–6 per stipe.	6.5–12 × 2.0–3.0 μm, 1–5 per metula, ampulliform to acerose, tapering into very thin neck.	2.0–2.5 × 1.7–2.0 μm, subglobose to ellipsoidal, smooth.	Absent	[16]

It also displays restricted growth on different media, long stipes, biverticillate penicilli, acerose phialides, and globose to subglobose or ellipsoidal smooth-walled conidia. It does not produce a sexual morph on any of the media tested, which is consistent with other species in ser. *Diversi*. Colonies of *T. marinus* on MEA (8–16 mm) and OA (5–8 mm) are notably smaller than those of other species within the series *Diversi.* When cultivated on YES, CZA, and MEA media, *T*. *marinus* exhibits a distinct clear zone resembling soluble pigment diffusion around the colony, distinguishing it from other species in the series. Exudate appears as small yellow droplets on CZA, whereas *T*. *albisclerotius* and *T*. *clemensii* produce as small clear droplets on OA and MEA, respectively [10, 66]. Unlike other species in the series *Diversi*, which typically grow on CREA within 7 days, *T*. *marinus* requires approximately 14 days for visible growth and subsequently produces acid, whereas *T*. *cystophilus* and *T*. *tianshanicus* do not grow on CREA [16, 65]. Small yellow droplets are produced as exudate exclusively on CZA, with no colored pigment observed on any of the media tested. A clear zone of metabolic diffusion around the colony is present on YES, CZA, and MEA. The conidiophore of *T*. *marinus* is unbranched, distinguishing it from *T*. *albisclerotius* [10] and *T*. *clemensii* [66]. Additionally, its conidiophore is smooth to finely roughened, unlike other species in ser. *Diversi* that have smooth walled stipes. The species most closely related to *T*. *marinus* are *T*. *cystophilus* and *T*. *tianshanicus* with distinguishing differences summarized in Table (2).

In pairwise nucleotide comparisons without gaps between *T. marinus* (SUMCC 15014) and *T. cystophilus* (CGMCC40002), there was a nucleotide difference of 1.6% (9/564 bp) in ITS, 0.8% (3/400 bp) in *BenA* and 0.5% (4/900 bp) in *RPB2* genes; and between *T. marinus* and *T*. *tianshanicus* (CGMCC 3.28741), there was a nucleotide difference of 1.3% (7/560 bp) in ITS, 0.7% (3/436 bp) in *BenA* and 0.5% (5/1050 bp) in *RPB2* genes. These nucleotide differences, together with the combined LSU–ITS–BenA–RPB2 phylogenetic, morphological, and cultural evidence, support the recognition of *T*. *marinus* as a distinct species.

**Fig. 2:**
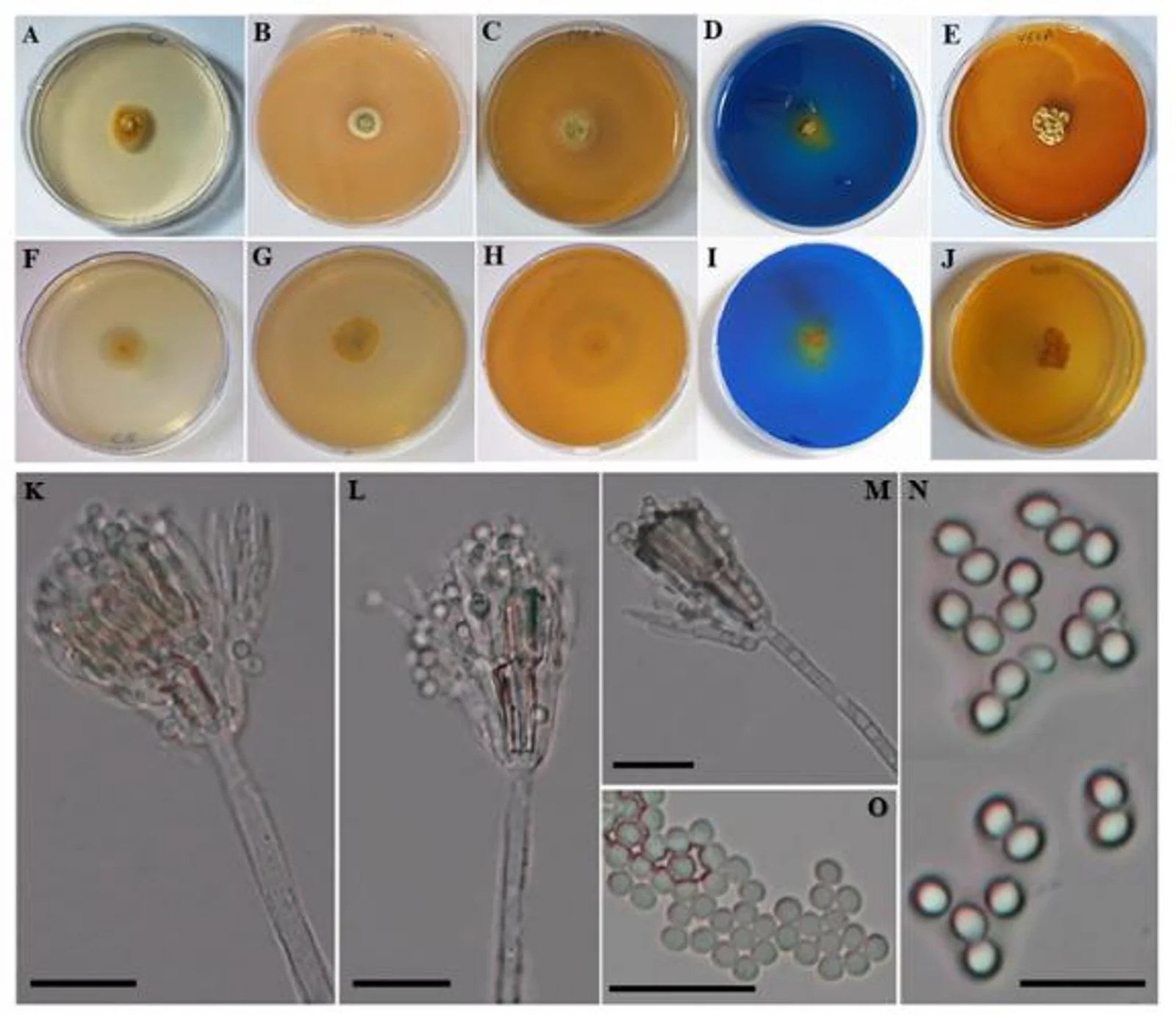
*Talaromyces marinus* (SUMCC 15014, ex-type). **A**–**E.** Colony color from left to right CZA, PDA, MEA, CREA and YES. **F**–**J.** Reverse color from left to right CZA, PDA, MEA, CERA and YES. **K**–**M.** Symmetrical biverticillate penicilli, appressed metulae and acerose phialides. **N**,** O.** Conidia. Bars: K–M = 10 μm; **N**, **O** = 5 μm

### Enzymatic activity FIG. 3A–D

The ability of *T. marinus* to produce various enzymes has been studied and screened for 13 extracellular enzymes on solid media. Table (3) summarizes the enzymatic activities of this new species. *Talaromyces marinus* efficiently produces asparaginase, xylanase and cellulase, while it produces gelatinase and tannase moderately. It also shows positive results for chitinase. Conversely, it does not produce lipase, inulinase or tyrosinase, but it efficiently generates laccase, ligninase, lignin peroxidase, and manganese peroxidase. Therefore, further quantitative studies on its enzymatic production and other secondary metabolites are highly recommended.

**Fig. 3:**
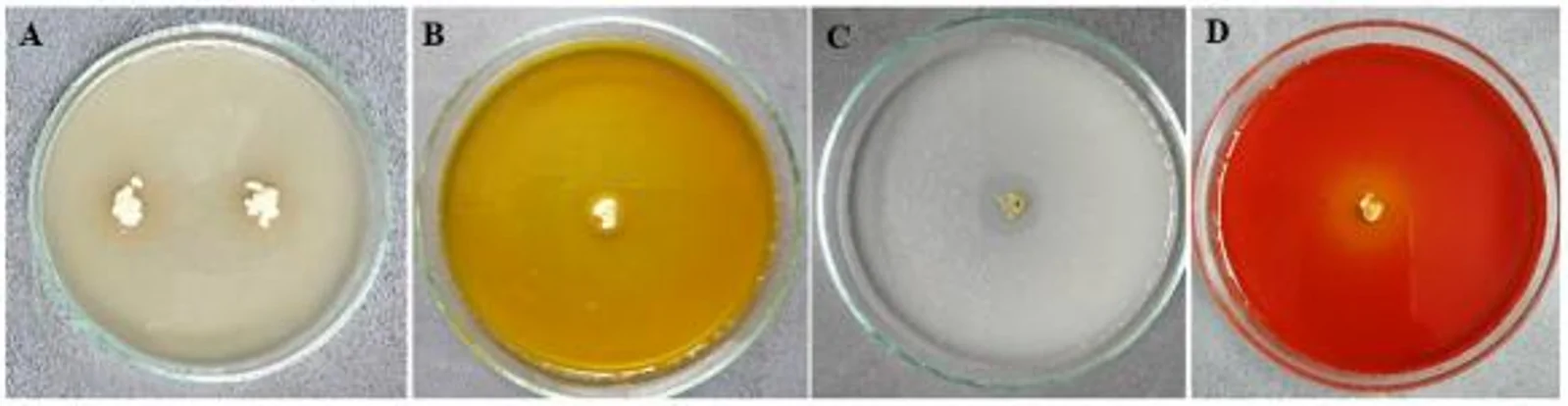
Testing enzymatic activities of *Talaromyces marinus*. **A**. Moderate production of gelatinase, **B**. Positive production of manganese peroxidase, **C**. Clear zone indicating efficient production of xylanase, **D.** Efficient production of L-asparaginase

**Table 3:** Qualitative enzymatic activities exhibited by *Talaromyces marinus*

Tested enzyme	Production
Asparaginase	+++
Cellulase	+++
Chitinase	+
Gelatinase	++
Inulinase	-
Laccase	+++
Ligninase	+++
Lignin peroxidase	+++
Lipase	-
Manganese peroxidase	+++
Tannase	++
Tyrosinase	-
Xylanase	+++

(+++) good production

(++) moderate production

(+) less production

(-) no production

## Discussion

The genus *Talaromyces* is a taxonomically and industrially significant group that has a substantial impact on daily life, particularly in the food industry, medical field, and biotechnology [2]. Its impact aroused significant interest in its taxonomy. Consequently, the number of newly described *Talaromyces* species has markedly increased in recent years [9, 16].

Through ongoing research, *T. marinus* is introduced as a new species to science based on morphological and phylogenetic evidence. Multi-locus phylogenetic analysis placed the new taxon, *T. marinus* in ser. *Diversi* within *Talaromyces* sect. *Trachyspermi* and clustered with *T. cystophilus* and *T*. *tianshanicus*. The ITS, *BenA* and *RPB2* phylograms also consistently show that the new taxon is closely related to *T. cystophilus* and *T*. *tianshanicus* with strong statistical support. Future studies incorporating additional strains, broader geographic sampling, and additional molecular markers may further improve our understanding of species boundaries within this lineage. *Talaromyces marinus* is a saprophytic species isolated from intertidal decaying woody samples of *A. marina* collected from marine environments in Egypt, while *T*. *cystophilus* is a nematode parasite isolated (as *T*. *cystophila*) from *Heterodera zeae* cyst in Guangxi, China [65], and *T*. *tianshanicus* was identified from soil sample collected from Western Tian Shan Mountains in Uzbekistan [16].

Several species of *Talaromyces* have been reported to produce diverse bioactive compounds, including anticancer, antimicrobial, enzymes, natural colorants, antiproliferative and antioxidative agents [19, 20, 23–25, 27, 28]. Enzymes produced by members of the genus *Talaromyces* are used in various industries, including food, paper, textiles, and biomedicine [68].

Different species of *Talaromyces* have been identified as good sources of specific enzymes. Although enzymatic studies within this specific lineage *Talaromyces* section *Trachyspermi* remain relatively limited compared to other sections of the genus, studies demonstrated the production of a broad spectrum of extracellular enzymes, including amylases, cellulases, proteases, and lipases [2, 34]. Most of the studies remain limited to selected species, and enzyme production within section *Trachyspermi* is still largely supported by qualitative or strain-specific investigations.

The diverse enzymatic capabilities of the *Talaromyces* spp. reflect their ecological adaptation and their saprophytic lifestyle. For example, *T. stipitatus* produces feruloyl esterases [69], *T. flavus* produces a thermophilic glucoamylase [70], and *T. versatilis* strain PF8 produces endo-1,3(4)-β-glucanase (3-(1–3;1–4)-β-d-glucan 3(4)-glucanohydrolase enzyme [71]. *Talaromyces marneffei*, *T*. *trachyspermus*, *T*. *barcinensis* and *T*. *ucrainicus* have been found capable of producing cellulolytic and pectinolytic enzymes [72, 73]. *Talaromyces emersonii* has also been shown to produce a broad range of enzymatic activities relevant to the hydrolysis of cellulose, hemicellulose and pectin [74]. Pasqualetti et al. [55] reported that only 11 of the 28 fungal species (39%) isolated from marine habitats possessed chitinolytic activity, highlighting the relatively limited distribution of chitin-degrading capability among marine fungi. *Talaromyces marinus* demonstrated the ability to produce cellulase and chitinase, that may be utilized in the synthesis of high-value chitin derivatives (chito-oligosaccharides) or in the degradation of chitin-rich materials such as leftovers from the shrimp or crab industries. It was also found to produce a notable amount of xylanase like *Talaromyces amestolkiae* [75], making it a potential source of xylanase.

L-asparaginase derived from marine sources presents a prospective avenue for clinical and food application due to its unique architectures, reduced molecular weights, and strong substrate selectivity [76]. Asparaginase is produced by a wide range of microorganisms, including fungi [77]. Fifteen asparaginase-producing fungal strains including *Talaromyces* sp. was isolated by Theantana et al. [53]. The new species, *T*. *marinus*, demonstrates a strong capacity to produce the asparaginase enzyme, making it a promising source of L-asparaginase.

Laccase offers significant advantages in environmental applications due to its natural secretion and high stability outside of cells. Rohrmann & Molitoris [78] were the first to report a marine fungus that produce considerable amounts of laccase when cultivated in a saltwater medium with peptone as the nitrogen source. Among forty fungi isolated from decomposing mangrove wood, three exhibited positive laccase activity when grown with guaiacol [59]. Laccase, manganese peroxidase, and lignin peroxidase lack substrate specificity, enabling the producer to degrade lignin and a variety of other xenobiotics, including industrial-colored wastewaters [57]. *Talaromyces marinus* does not produce lipase but efficiently generates laccase, ligninase, lignin peroxidase, and manganese peroxidase, making it a potential source of these enzymes for various industries. Upon screening more than 400 strains of marine yeast from diverse marine environments, only a few strains were able to release significant amounts of inulinase [79], similarly, *T. marinus* showed no activity in Inulinase production. However, it possesses moderate capacity to produce tannase and gelatinase.

Tyrosinase catalyzes the oxidation of o-diphenols into o-quinones and the orthohydroxylation of monophenols to o-diphenols [80]. Among twenty fungal isolates tested for tyrosinase activity, only two produced the enzyme [81]. Similarly, *T*. *marinus* was unable to synthesize tyrosinase enzyme. *Talaromyces marinus* has shown promising preliminary results in producing various enzymes that made it allied with other results of species under sec. *trachyspermi*, making it a new potential source for these enzymes in different industries. However, further quantitative studies are needed to configure precisely the amount of these enzymes.

## Conclusion

A new marine saprophytic fungal species, *T. marinus* is introduced to science based on cultural, morphological and phylogenetic evidence. It is isolated from intertidal decaying woody samples of *A. marina* collected from a mangrove stands in the Wadi El-Jimal area along the Red Sea coast of Egypt. Multi-locus phylogenetic analysis placed the new taxon in *Talaromyces* sect. *Trachyspermi* ser. *Diversi* and clustered with *T*. *cystophilus* (as *T*. *cystophila*) and *T*. *tianshanicus*. It exhibits the general morphological diagnostic characteristics of the *Talaromyces* section *Trachyspermi* ser. *Diversi*. It is characterized by restricted growth on different media, long stipes, biverticillate penicilli, the shortest acerose phialides in series *Diversi*, globose to subglobose or ellipsoidal, smooth-walled conidia, acid production, and soluble pigments. Qualitative enzymatic capabilities of *T. marinus* were studied and revealed that it has the ability to produce cellulase, chitinase and it has a strong capacity to produce the asparaginase enzyme. *Talaromyces marinus* does not produce lipase, inulinase or tyrosinase but efficiently generates laccase, ligninase, lignin peroxidase, and manganese peroxidase, making it a potential source of these enzymes. The promising capabilities of *T. marinus* to produce various enzymes, making it a clean and renewable source for these enzymes in different industries. However, further quantitative studies are needed.

## Supplementary Information

Supplementary Material 1.

Supplementary Material 2.

Supplementary Material 3.

## Data Availability

The ITS region, LSU, **BenA**, and **rpb2** gene sequences of **Talaromyces marinus** have been deposited at Gen-Bank under the accession numbers PX683786, PX683787, PX694710, and PX694709. https://www.ncbi.nlm.nih.gov/nuccore/PX683786.1; https://www.ncbi.nlm.nih.gov/nuccore/PX683787; https://www.ncbi.nlm.nih.gov/nuccore/PX694710; https://www.ncbi.nlm.nih.gov/nuccore/PX694709.
